# A narrative review of nosology and the concept of schizophrenia: criticism and proposal

**DOI:** 10.31744/einstein_journal/2025RW1131

**Published:** 2025-02-14

**Authors:** Ricardo Abreu Feijo de Mello, Ary Gadelha, Larissa Leal Freitas, Vitoria Fernandes Sant’Ana, Marcelo Feijó Mello

**Affiliations:** 1 Faculdade Israelita de Ciências da Saúde Albert Einstein Hospital Israelita Albert Einstein São Paulo SP Brazil Faculdade Israelita de Ciências da Saúde Albert Einstein, Hospital Israelita Albert Einstein, São Paulo, SP, Brazil.; 2 Faculdade de Ciências Médicas Santa Casa de São Paulo São Paulo SP Brazil Faculdade de Ciências Médicas da Santa Casa de São Paulo, São Paulo, SP, Brazil.; 3 Escola Paulista de Medicina Universidade Federal de São Paulo São Paulo SP Brazil Escola Paulista de Medicina, Universidade Federal de São Paulo, São Paulo, SP, Brazil.

**Keywords:** Schizophrenia, Psychiatric disorders, Psychopathology, Psychotic disorders

## Abstract

Schizophrenia diagnostics have evolved to adapt to clinical needs and scientific advances, and the current denominations emerged at the beginning of the twentieth century. Most problems arise while integrating clinical experiences, based on historical psychopathological descriptions, with emerging translational neuroscience research. This study aimed to evaluate the state-of-the-art critics of the current schizophrenia concept and their recommendations for new concepts. We performed a narrative review of the literature and searched for studies published in English in PubMed in the last 2 years which discussed the diagnosis of schizophrenia. Two authors independently selected the studies after analyzing the abstracts. Subsequently, studies were selected for this review by consensus. Twenty-six studies were selected, and all authors, except two, had restrictions on the current categorical model for the diagnosis of schizophrenia owing to the heterogeneity of symptomatology and high frequency of comorbidity. Eight studies proposed changes to the concept of schizophrenia. The central proposition was to adopt psychotic syndrome as a core feature instead of the current concept of schizophrenia. We synthesize these proposals using psychosis as a spectrum that includes schizophrenia as a more severe case at the end of the spectrum.

## INTRODUCTION

Evidence shows a high impact of the diagnosis of schizophrenia on patients. Hjorthøj et al. described that patients with schizophrenia lost 13-15 years of life and presented a risk of suicide twenty-two times over.^1^ Moreover, patients with schizophrenia have not aggregated the health improvement benefits received by the general population, resulting in a higher mortality gap over the last three decades. In a systematic review, Saha et al.^2^ found that patients with schizophrenia had 2.5 times higher odds of dying than those of the same age in the general population. A better understanding of the underlying neurobiology is usually suggested as a significant challenge for better diagnostic and treatment tools. In contrast, other authors pointed to a high stigma or lack of health policies to apply current best-evidence practices. A central discussion connecting these topics is the definition of schizophrenia.

Schizophrenia is a nosological entity that reflects the challenges, advances, and drawbacks of the current diagnostic criteria in psychiatry.^3^ Most clinicians and researchers agree that schizophrenia is a prototype diagnosis of a primary psychotic disorder tied to the underlying idea of disruption in the sense of reality. Several problems have emerged from this perspective.^4^ Some of these can be attributed to clinical perspectives, such as the thresholds for impairment, symptoms required, and duration of illness. Others have emerged more recently, with difficulties in converging neurobiological findings to validate clinicians’ assumptions about the disease. More importantly, the slow progress in new treatments and diagnostic methods requires a review of the current definitions, as reported by Gama Marques et al.^5^

### Historical aspects

The current criteria are consensual and anchored in classical descriptions. During the nineteenth century, the authors^6-10^ described severe psychiatric disorders as catatonia, hebephrenia, and paranoia, respectively. At the end of the century, Kraepelin found a common outcome for all of them and called it dementia praecox.^11,12^ A review of Kraepelin’s work by Kendler showed that the author refined his theories. Kraepelin et al. did not characterize dementia praecox because of its chronic nature and poor prognosis. They recognized the episodic course, especially catatonia, and a substantial proportion of patients evolved with moderate deterioration and some with complete recovery. They reinforced the importance of bizarre delusions and symptoms of passivity described later as the “Schneiderian’s symptoms.”

At the beginning of the twentieth century, Bleuler and Maatz et al^13,14^ introduced the concept of Schizophrenia, looking for a clinical unity that allowed a scientific description and classification. Bleuler proposed that schizophrenia is caused by disturbed cognitive processing characterized by division or fragmentation of the association process.

Later in the same century, Schneider described the longitudinal and transversal dimensions of schizophrenia using a pragmatic approach, incorporating hallucinations and delusions, which were considered accessories by Kraepelin^12^ and Bleuler^13^ as central to the disease. The current schizophrenia diagnostics have incorporated Schneider’s criteria**.**

The current diagnostic system emphasizes the positive and negative symptoms first described by Reynolds^15^and Jackson.^16^The first correlates with a denial of vital properties and the former with an imbalance in brain areas. Crow was the first to link these negative and positive symptoms as validators of premorbid functioning, responses to treatment, and neuropsychological findings. The positive characteristics were associated with better premorbid functioning, acute onset, normal cognition, and a reasonable response to treatment. The negative characteristics were associated with early disease onset, impaired cognition, and poor response to treatment. The deficit and non-deficit forms of schizophrenia have been conceptualized and validated.^17,18^

Over the years, clinicians have suggested different perspectives, mainly based on observations of institutionalized patients. There is also a clear impact of mainstream theories when reviewing the changes in definitions. In the first two versions of the Diagnostic and Statistical Manual (DSM) of the American Psychiatric Association, the diagnostic criteria were based mainly on psychoanalytic perspectives. This approach reduced the generalizability by focusing on subjectivity and individual experiences. By the end of the ‘60s, the United States-United Kingdom project revealed the need for more standardized criteria for reliability between sites and interviewers.^19^ Reliability was the main guiding force in reviewing the final diagnostic criteria.

### The official diagnostic definitions

The current categorical definitions of schizophrenia proposed by the DSM and International Classification of Diseases have been criticized for their poor association with specific underlying neurobiological mechanisms and high clinical heterogeneity, limiting prognostic predictions and treatment outcomes.^20-26^ The difficulties in converging clinical experience through historical psychopathological descriptions to the translational neuroscience approach remain challenging. Over the years, proposals and criticisms have been developed to address the nosological problems of schizophrenia. Herein, we propose evaluating the state-of-the-art critics of the current schizophrenia concept and their recommendations for new concepts.

We conducted a narrative review of relevant literature. In June, 2022, the authors searched PubMed using the terms (“schizophrenia’ OR ‘schizophrenic disorder”) AND Nosology OR ‘Diagnostic Criteria’ published over the previous 2 years. Two authors (RAFM and MFM) separately analyzed the abstracts and selected studies using the following inclusion criteria: reflections on the diagnosis of schizophrenia written in English, and a proposal for future research. The studies were also selected from the bibliographies of the selected studies. Two authors systematically extracted data from the selected studies. We analyzed the results and suggested suggestions for further studies in nosology.

We followed the steps suggested by the “Scale for the Assessment of Narrative Review Articles (SANRA),” to determine the quality of the narrative review.^27^Baethge et al., from the Department of Psychiatry and Psychotherapy of the University of Cologne Medical School, developed the SANRA, a brief critical appraisal tool for the assessment of non-systematic articles, as a tool for improving the quality of narrative reviews. SANRA was developed and improved by this group from Cologne, and the current version has a 6-item rating from 0 (low standard) to 2 (high standard), and 12 being the maximum score.^28^ The sum score of the scale is intended to measure the construct “quality of a narrative review article,” covering the topics: explanation of the review’s importance (item 1) and statement of the aims (item 2) of the review, description of the literature search (item 3), referencing (item 4), scientific reasoning (item 5), and presentation of relevant and appropriate endpoint data (item 6). SANRA’s feasibility, inter-rater reliability, homogeneity of items, and internal consistency are sufficient for a scale of 6 items.^27^

### Current schizophrenia concepts and new recommendations

Twenty-six studies were selected, and the content related to opinions on schizophrenia and their proposals are summarized in table 1. The table lines refer to the authors and year of publication, main criticisms of schizophrenia diagnosis, strategies to improve diagnosis, and proposals to change the concept.

**Table 1:** Relevant points in papers that critique the current concept of schizophrenia

	Authors (Year)							
	Gordon et al (2022)^(40)^	Martínez-Alés et al (2022)^(41)^	Tamminga et al (2022)^(30)^	Owen et al (2022)^(31)^	Meyer-Lindenberg et al (2022)^(48)^	Zick et al (2022)^(32)^	Kotov et al (2022)^(33)^	Keshavan et al (2022)^(42)^
On the current schizophrenia concept	Needs to change	Criticized the concept	Did not criticize the concept	Schizophrenia is a developmental disorder that affects many brain functions	Reinforced the lack of clear barriers between psychiatric diagnoses based on common genetic risks	Schizophrenia is a group of disorders with an infinite combination of genetic, molecular, synaptic, micro-, and macro-circuit alterations with metacognitive and environmental vulnerabilities, manifesting with a high level of individual variation	The low reliability between evaluators and the temporal stability of the schizophrenic disorder diagnosis proposed by the DSM were due to the arbitrary limits between categorical diagnoses applied to psychopathology that works in a continuum	Criticized the concept as they considered it inadequate to research using biological markers
Proposal	the use of the RDoC associated with a Bayesian data inference approach to test the clinical utility of new patient characterizations	Need for advances in neurobiology and genetics, besides understanding cultural variations	Utilization of biomarkers as auxiliaries to conventional diagnosis to enhance treatment	To study the correlations of these dynamic brain alterations with genetic variants highly expressed in the same brain regions	Has no proposal	Change the name schizophrenia to disorders of the psychotic spectrum due to the stigma carried by the name and by the highly heterogeneous clinical presentations	An alternative to the schizophrenia diagnosis is based on a psychopathology continuum (HiTOP). The diagnostic proposal had two fundamental dimensions in psychotic experiences: psychoticism and detachment.	Considered the use of psychosis as a syndrome. The authors proposed using biomarkers related to signal-to-noise ratio (SNR) as a measure of brain efficiency in stimuli apprehension, the basis of a psychotic experience
	**Authors (Year)**							
	**Waddington et al (2022)^(43)^**	**Kelly et al (2022)^(34)^**	**Tandon et al (2022)^(50)^**	**Nasrallah et al (2022)^(35)^**	**Murray et al (2022)^(44)^**	**Gooding (2022)^(36)^**	**Reichenberg et al (2022)^(57)^**	**Smoller et al (2022)^(37)^**
On the current schizophrenia concept	Reinforced the concept of primary psychoses in the nineteenth-century	Criticized the concept and the delay in deconstructing the term	Believes that the utilization of schizophrenia as a diagnosis leads to heterogenous case presentations and pathophysiological differences	The state-of-the-art knowledge shows that hundreds of genetic factors can disrupt the development of the brain at various stages of life, starting at the fetal period, alongside non-genomic factors mediated by epigenetic changes, leading to functional and structural neurodevelopment aberrations	Cites considerable evidence showing the precarity of the concept	Considers schizophrenia as a disorder of neurodevelopment mediated by genetics and should be understood as a group of diseases with diverse etiologies that require different treatments	Reinforced the notion that schizophrenia is a dynamic process that initiates before the beginning of clinical symptoms presentation	The concept is an umbrella with a collection of hereditary components
Proposal	Has no proposal	Has no proposal	Schizophrenia as a series of pathological disorders under the same name	Urges the schizophrenia syndrome reconceptualization	Has no proposal	Has no proposal	The disease would be the final stage of this neurodevelopmental process	Believes that genetics is a way to refine the schizophrenia construct

Most studies underscored the need to change or refine the current definition of schizophrenia; however, three^29-31^were not critical of the concept. Eight studies suggested changes to the name; although there was no consensual name, the terms psychotic spectrum and disorders were repeated.^32-39^

Most authors^30,32-34,38-46^ considered that the DSM concept had unclear limitations for other psychiatric disorders, creating difficulties in research with a clear impact on clinical activities. Refining the research methods using advanced genetics^29,37,47,48^ alone or associated with other biomarkers will improve the understanding of underlying mechanisms that deal with the research and clinical difficulties. Six studies indicated that understanding schizophrenia as a spectrum syndrome of neurodevelopmental origins, with a diversity of genetic variations leading to wide variation in the functional and structural functions of the brain, is a way to overcome these difficulties.^31,32,35-37,49^

Some authors have proposed conceptual changes, such as the “Hierarchical Taxonomy Of Psychopathology^33^using translational psychopathology. Gordon et al.^40^ proposed the use of the National Institute of Mental Health Research Domain Criteria^26^ associated with a Bayesian data inference approach.

The main findings of this study regarding the present diagnostic construct of schizophrenia are: 1) the use of a dimensional diagnostic system considering genetic and other biomarkers, and 2) to change the term schizophrenia by terms referring as a psychoses developmental-related syndrome.

The criticisms of recent literature search focused on three main topics: 1) lack of a core feature, 2) overlap of symptoms with other disorders, and 3) low diagnostic reliability. The strategies to improve diagnosis were as follows: 1) use of biomarkers, 2) dimensional approaches, and 3) multicultural validation.

Only eight authors proposed a change in the term schizophrenia. Some proposed changing the name to “Psychotic Spectrum Illness,” concluding that patients not only suffer not from a syndrome but also from a conglomerate of infinite combination of factors, such as genetics, molecular, synaptic, micro- and macro-circuitry, cognitive, metacognitive, and environmental vulnerabilities.^32-34,38,42-44,50^ A similar idea was considered by van et al^4^ that schizophrenia is a fraction of prognostically reserved patients with complex multidimensional psychotic syndrome; it is age-dependent (>10 years old), and its expression is measurable in the general population. The correlated symptoms include psychotic experiences (hallucinations and delusions), motivational impairment (avolition and amotivation), affective dysregulation, and alterations in information processing (cognitive impairment). Heritability indicates a strong genetic influence, as estimated by twin studies and gene-environment interactions.

The proposition of changing the name of schizophrenia went beyond the academic world. Social movements led by patients and their families were organized to ask for the elimination of names due to harm and stigmatization. This movement occurred in countries such as the UK, US, and Canada.^51^ In the academic field, the psychiatrists followed eminent psychiatrists such as Murray and van Os, who proposed the abolition of the term.^52,53)^

Japan officially renamed schizophrenia in 2005 as a two-decade process initiated by a social movement until the Japanese Society of Psychiatry and Neurology replaced it with the term ‘integration dysregulation syndrome. The new term refers to the stress-vulnerability model of the disorder, underlining that it is treatable, and recovery is possible with a combination of advanced pharmacotherapy and psychosocial intervention. The Korean Neuropsychiatric Association and Korean Society for Schizophrenia Research renamed ‘schizophrenia’ as ‘attunement disorder’ in 2011.^51^

The Italian Association of Psychiatry surveyed how psychiatrists, service users, and family members perceived the term schizophrenia and if they considered a name change as a helpful option to overcome the stigma attached to it. For all samples, 41.5% found the term inappropriate, 67.6% stigmatized it, and 72.3% advocated for a name change. Among psychiatrists, 57% reported that schizophrenia was inappropriate, 70% considered the term stigmatizing, and 71% favored a name change. Similarly, 56% of service users and 71% of family members found schizophrenia to be a stigmatizing term, and 75% and 77% advocated a name change, respectively. Conflicting results were found on possible alternative terms: psychiatrists proposed a wide range of possible options, most of which referred to the term ‘psychosis’ (53%), whereas users and family members preferred terms referring to the broad category of ‘mental health suffering.’^54^

Mesholam-Gately et al.^55^developed a project to collect opinions on possible name changes for schizophrenia from a broad, diverse sample of stakeholders, including those with lived experience of mental illness, family members, clinicians, researchers, government officials, and the general public. They found that most respondents (74.1%) favored a name change for schizophrenia. Most participants (71.4%) reported the name stigmatizing. Of the proposed alternate names, those with the most support included “Altered Perception Syndrome,” “Psychosis Spectrum Syndrome,” and “Neuro-emotional Integration Disorder.” The survey findings provided strong support for renaming schizophrenia. Most expressed hope that a name change would reduce the stigma and discrimination.

The replacement using terms such as psychoses or psychotics requires a prior definition. The official nosology defines psychosis as the presence of delusions or hallucinations. However, such a definition can be misleading; hence, asking about delusional content may lead to a false identification of psychosis in patients with cultural or personal interpretations that are not psychotic from a deeper understanding, as defined from a phenomenological perspective. From a functional perspective, psychosis is considered a disruption of the self, leading to subjective fading and impairment of the ability to distinguish reality from imagination, with the emergence of either delusions or hallucinations. Psychosis is an emergent syndrome associated with profound alterations in the cognitive and affective functions. Psychotic symptoms can manifest under different circumstances, ranging from isolated and non-pathological symptoms in the general population to severe manifestations such as complete disaggregation, indicating the existence of different pathogenic mechanisms. Most authors agree to center on a new concept of psychosis; however, an objective consensual definition is still lacking.

Murray et al^44^ of the Kings College Institute of Psychiatry published their article citing the famous British nosologist Ian Brockington’s strong criticism of “schizophrenia as an idea whose essence is equivocal, a nosological category without natural boundaries, and a barren hypothesis. Such a blurred concept is not a valid object of scientific inquiry; it is a cloak for ignorance and exposes psychiatry to ridicule. As a model of psychosis, this oversimplification serves neither the interests of scientists nor those of patients. It is time to abandon this concept, because of the history of semantic wrangles.” Thirty years later, the critique remains true.

In an editorial, Tandon et al^50^ comments on a survey of 50 schizophrenia experts, where a significant majority of them advocated the retention of the schizophrenia construct, although some did so with restrictions. Many researchers consider this construct clinically useful. Some suggested replacing it with a broader construct of primary psychosis or psychosis spectrum disorder, whereas others argued for its elimination. Alternative constructs include a hierarchical psychopathological model and a biotype architecture.^30,42^ Many authors have emphasized the need to view this construct as a syndrome.^50^

We agree with the current limitations of the schizophrenia construct in research and the stigma carried by patients with schizophrenia and their families. The understanding that their symptoms result from multiple factors interfering in the neurological and psychological developments, representing a more severe point of a syndrome or a spectrum illness, is a tendency to deal with the difficulties. Radical changes in psychopathological tools adapted to advances in neuroscience will facilitate translational communication between basic and clinical sciences.

### Limitations

Most authors were from North America or Europe, leading to a possible bias in patient profiles compared to other regions worldwide. Most authors have not considered the issues related to minorities or cultures. Different studies using replicable methods have shown consistent and dose-dependent associations between psychotic disorders and minority groups, relative to integrating these groups into the community, and demonstrating the impact of discrimination and social marginalization. Fonseca et al.^56^reinforced the lack of diversity in genetic and environmental backgrounds, which weakens the generalizability and clinical applicability of research findings to psychotic disorders. Notably, Latin Americans have generally been neglected in genetic studies, comprising less than 2% of genome-wide associated study samples. The authors considered the period for the literature to be after the publication of a special issue in a specialized journal that invited relevant researchers from different countries to discuss the theme of the present review, including possible studies published as reactions to the special issue. However, this is a limitation of this study. The strength of this study is that it uses a narrative review, which is more suitable for our question, as we were not looking for quantitative data; however, we tried to enhance the quality of our review using validated parameters, such as the SANRA scale.

### Final comments

Despite the evidence of dissatisfaction with the term schizophrenia among health service users, their families, and mental health workers, including psychiatrists and researchers, and the claim for its change, the movements to realize it are discreet. The proposals generally focus on discrete views that depend on the proponent’s interests, either in social or research areas. Brainstorming to aggregate diversity and create a different concept, which is necessary for a paradigm change, is absent.

## References

[1] Hjorthøj C, Stürup AE, McGrath JJ, Nordentoft M (2017). Years of potential life lost and life expectancy in schizophrenia: a systematic review and meta-analysis. Lancet Psychiatry.

[2] Saha S, Chant D, McGrath J (2007). A systematic review of mortality in schizophrenia: is the differential mortality gap worsening over time?. Arch Gen Psychiatry.

[3] McGrath JJ, Wray NR (2022). Seven short reflections on the notion of schizophrenia. Schizophr Res.

[4] van Os J, Kenis G, Rutten BP (2010). The environment and schizophrenia. Nature.

[5] Gama Marques J, Ouakinin S (2021). Schizophrenia-schizoaffective-bipolar spectra: an epistemological perspective. CNS Spectr.

[6] Starkstein SE, Goldar JC, Hodgkiss A (1995). Karl Ludwig Kahlbaum's concept of catatonia. Hist Psychiatry.

[7] Kahlbaum KL (1874). Die Katatonie oder das Spannungsirreseins.

[8] Jäger M, Becker T, Wigand ME (2018). Hebephrenie - ein brauchbares psychopathologisches Konstrukt?. Nervenarzt.

[9] Hecker E (1871). Die Hebephrenie. Ein Beitrag zur klinischen Psychiatrie. Virchows Arch.

[10] Heinroth JC (1818). Lehrbuch der Störungen des Seelenlebens oder der Seelenstörungen und ihrer Behandlung.

[11] Kendler KS (2021). Kraepelin's Final Views on Dementia Praecox. Schizophr Bull.

[12] Kraepelin E (1893). Psychiatrie: Ein kurzes Lehrbuch für Studirende un Aerzte.

[13] Bleuler E (1911). Dementia praecox, oder Gruppe der Schizophrenien.

[14] Maatz A, Hoff P, Angst J (2015). Eugen Bleuler's schizophrenia-a modern perspective. Dialogues Clin Neurosci.

[15] Reynolds JR (1858). On the reference of the pathology of convulsions with special reference of those children. Liverport Med Clin J.

[16] Jackson J (1889). On postepileptics states: a contribution to comparative studies of insanities. J Ment Sci.

[17] Carpenter WT, Heinrichs DW, Wagman AM (1988). Deficit and nondeficit forms of schizophrenia: the concept. Am J Psychiatry.

[18] Crow TJ (1985). The two-syndrome concept: origins and current status. Schizophr Bull.

[19] Kendell RE, Cooper JE, Gourlay AJ, Copeland JR, Sharpe L, Gurland BJ (1971). Diagnostic criteria of American and British psychiatrists. Arch Gen Psychiatry.

[20] Mataix-Cols D, Rosario-Campos MC, Leckman JF (2005). A multidimensional model of obsessive-compulsive disorder. Am J Psychiatry.

[21] Trull TJ, Durrett CA (2005). Categorical and dimensional models of personality disorder. Annu Rev Clin Psychol.

[22] Aragona M (2006). A bibliometric analysis of the current status of psychiatric classification: the DSM model compared to the spectrum and the dimensional diagnosis. Giorn Ital Psicopat.

[23] Lochner C, Stein DJ (2006). Does work on obsessive-compulsive spectrum disorders contribute to understanding the heterogeneity of obsessive-compulsive disorder?. Prog Neuropsychopharmacol Biol Psychiatry.

[24] Helzer JE, Kraemer HC, Krueger RF (2006). The feasibility and need for dimensional psychiatric diagnoses. Psychol Med.

[25] Adam D (2013). Mental health: on the spectrum. Nature.

[26] Insel TR (2014). The NIMH Research Domain Criteria (RDoC) Project: precision medicine for psychiatry. Am J Psychiatry.

[27] Baethge C, Goldbeck-Wood S, Mertens S (2019). SANRA-a scale for the quality assessment of narrative review articles. Res Integr Peer Rev.

[28] Baethge CG, Mertens S, Goldbeck-Wood S (2017). A scale for the assessment of nonsystematic review articles (SANRA).

[29] DeLisi LE (2022). Redefining schizophrenia through genetics: A commentary on 50 years searching for biological causes. Schizophr Res.

[30] Tamminga CA, Pearlson G, Gershon E, Keedy S, Hudgens-Haney ME, Ivleva EI (2022). Using psychosis biotypes and the Framingham model for parsing psychosis biology. Schizophr Res.

[31] Owen MJ, Legge SE (2022). The nature of schizophrenia: as broad as it is long. Schizophr Res.

[32] Zick JL, Staglin B, Vinogradov S (2022). Eliminate schizophrenia. Schizophr Res.

[33] Kotov R, Jonas KG, Lian W, Docherty AR, Carpenter WT (2022). Reconceptualizing schizophrenia in the Hierarchical Taxonomy Of Psychopathology (HiTOP). Schizophr Res.

[34] Kelly DL, Buchanan RW (2022). Can the current schizophrenia construct endure?. Schizophr Res.

[35] Nasrallah HA (2022). Re-inventing the schizophrenia syndrome: the elusive "theory of everything". Schizophr Res.

[36] Gooding DC (2022). Brave New World: harnessing the promise of biomarkers to help solve the epigenetic puzzle. Schizophr Res.

[37] Smoller JW (2022). What can genetics tell us about the schizophrenia construct?. Schizophr Res.

[38] Gur RE (2022). Considering alternatives to the schizophrenia construct. Schizophr Res.

[39] Gureje O, Ojagbemi A (2022). Applicability and future status of schizophrenia as a construct in Africa. Schizophr Res.

[40] Gordon JA, Morris SE, Avenevoli S (2022). A framework for integration of dimensional and diagnostic approaches to the diagnosis of schizophrenia. Schizophr Res.

[41] Martínez-Alés G, Susser ES (2022). A useful construct to improve the lives of people with schizophrenia. Schizophr Res.

[42] Keshavan MS, Yassin W, Stone WS (2022). Conceptualizing psychosis as an information processing disorder: Signal, bandwidth, noise, and bias. Schizophr Res.

[43] Waddington JL, Nkire N, Russell V (2022). Schizophrenia vis-à-vis dimensional-spectrum concepts of psychotic illness: has an answer been 'hiding in plain sight'?. Schizophr Res.

[44] Murray RM, Quattrone D (2022). The Kraepelian concept of schizophrenia: dying but not yet dead. Schizophr Res.

[45] Sawa A, Yang K, Cascella NG (2022). Paradigm shift on the concept of schizophrenia that matches with both academic and clinical needs. Schizophr Res.

[46] McCutcheon RA, McGuire P (2022). Reinventing schizophrenia: the rules of the game. Schizophr Res.

[47] Chase-Lansdale PL, Owen MT (1987). Maternal employment in a family context: effects on infant-mother and infant-father attachments. Child Dev.

[48] Meyer-Lindenberg A, Hirjak D (2022). Schizophrenia as a categorical diagnosis: A view from the neural risk architecture. Schizophr Res.

[49] Cannon TD (2022). Psychosis, schizophrenia, and states vs. traits. Schizophr Res.

[50] Tandon R, Keshavan M, Nasrallah H (2022). Reinventing schizophrenia. Updating the construct. Schizophr Res.

[51] Lasalvia A (2018). Words matter: after more than a century 'schizophrenia' needs rebranding. BJPsych Adv.

[52] Murray R (2006). Schizophrenia term use 'invalid'. BBC News.

[53] van Os J (2016). "Schizophrenia" does not exist. BMJ.

[54] Lasalvia A, Vita A, Bellomo A, Tusconi M, Favaretto G, Bonetto C (2021). Renaming schizophrenia? A survey among psychiatrists, mental health service users and family members in Italy. Schizophr Res.

[55] Mesholam-Gately RI, Varca N, Spitzer C, Parrish EM, Hogan V, Behnke SH (2021). Are we ready for a name change for schizophrenia? A survey of multiple stakeholders. Schizophr Res.

[56] Fonseca L, Sena BF, Crossley N, Lopez-Jaramillo C, Koenen K, Freimer NB (2021). Diversity matters: opportunities in the study of the genetics of psychotic disorders in low- and middle-income countries in Latin America. Br J Psychiatry.

[57] Reichenberg A, Akbarian S (2022). Towards DSM 10: A bio-classification of developmental schizophrenia?. Schizophr Res.

